# Assessment of Carbon Sequestration Capacity of *E. ulmoides* in Ruyang County and Its Ecological Suitability Zoning Based on Satellite Images of GF-6

**DOI:** 10.3390/s23187895

**Published:** 2023-09-15

**Authors:** Juan Wang, Xinxin Wei, Shuying Sun, Minhui Li, Tingting Shi, Xiaobo Zhang

**Affiliations:** 1 State Key Laboratory for Quality Ensurance and Sustainable Use of Dao-di Herbs, National Resource Center for Chinese Materia Medica, China Academy of Chinese Medical Sciences, Beijing 100700, China; 2 School of Pharmaceutical Sciences, Changchun University of Chinese Medicine, Changchun 130117, China; 3 School of Life Sciences, Inner Mongolia University, Hohhot 010070, China; 4 Inner Mongolia Traditional Chinese & Mongolian Medical Research Institute, Hohhot 010010, China

**Keywords:** *E. ulmoides*, GF-6 WFV, red-edge band, random forest, net primary productivity, ecological suitability

## Abstract

*Eucommia ulmoides* Oliver. (*E. ulmoides*) is a species of small tree native to China. It is a valuable medicinal herb that can be used to treat Alzheimer’s disease, diabetes, hypertension, and other diseases. In addition, *E. ulmoides* is a source of rubber. It has both medicinal and ecological value. As ecological problems become increasingly prominent, accurate information on the cultivated area of *E. ulmoides* is important for understanding the carbon sequestration capacity and ecological suitability zoning of *E. ulmoides*. In previous tree mapping studies, no studies on the spectral characteristics of *E. ulmoides* and its remote sensing mapping have been seen. We use Ruyang County, Henan Province, China, as the study area. Firstly, using the 2021 Gao Fen-6 (GF-6) Wide Field of View (WFV) time series images covering the different growth stages of *E. ulmoides* based on the participation of red-edge bands, several band combination schemes were constructed. The optimal time window to identify *E. ulmoides* was selected by calculating the separability of *E. ulmoides* from other land cover types for different schemes. Secondly, a random forest algorithm based on several band combination schemes was investigated to map the *E. ulmoides* planting areas in Ruyang County. Thirdly, the annual NPP values of *E. ulmoides* were estimated using an improved Carnegie Ames Stanford Approach (CASA) to a light energy utilization model, which, in turn, was used to assess the carbon sequestration capacity. Finally, the ecologically suitable distribution zone of *E. ulmoides* under near current and future (2041–2060) climatic conditions was predicted using the MaxEnt model. The results showed that the participation of the red-edge band of the GF-6 data in the classification could effectively improve the recognition accuracy of *E. ulmoides*, making its overall accuracy reach 96.62%; the high NPP value of *E. ulmoides* was mainly concentrated in the south of Ruyang County, with a total annual carbon sequestration of 540.104835 t CM^−2^·a^−1^. The ecological suitability zone of *E. ulmoides* can be divided into four classes: unsuitable area, low suitable area, medium suitable area, and high suitable area. The method proposed in this paper applies to the real-time monitoring of *E. ulmoides*, highlighting its potential ecological value and providing theoretical reference and data support for the reasonable layout of *E. ulmoides*.

## 1. Introduction

**Figure 1:**
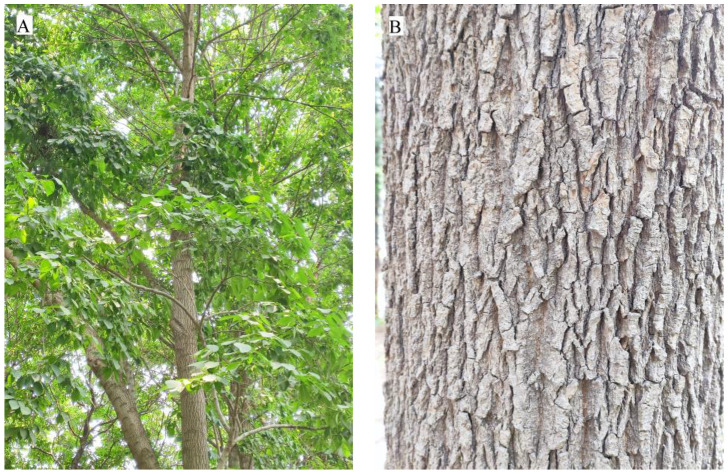
*E. ulmoides*. (**A**) *E. ulmoides* forest. (**B**) *E. ulmoides* parts of medicine: *E. ulmoides* Cortex.

People are becoming increasingly interested in medicinal plants as they become more health conscious. Eucommiae Cortex is the dried bark of *E. ulmoides* (Figure 1), a genus of Eucommia, a popular herb and health product in China, Japan, and Korea. Modern pharmacological studies have shown that *E. ulmoides* has been widely used to treat hypertension, osteoporosis, Alzheimer’s disease, diabetes, and sexual dysfunction. In addition, *E. ulmoides* is an endemic tree species that belongs to the second category of protected rare and endangered tree species in China [1], which is a high-quality natural rubber resource with great development potential in the world to solve the scarcity of rubber resources and an important tree species in maintaining ecological security and increasing carbon sinks. Ruyang County has a long history of *E. ulmoides* cultivation and is one of the main production zones of *E. ulmoides*. In December 2017, “Ruyang *E. ulmoides*” was approved by the Chinese Ministry of Agriculture and Rural Development as the title of the species under the “Geographical Indication of Agricultural Products”. Therefore, it is important to study the method of real-time monitoring of large zones of *E. ulmoides*, the assessment of carbon sequestration capacity, and its ecological suitability zone prediction.

In this context, remote sensing plays a key role in monitoring the range of medicinal plants. Currently, scholars have conducted studies related to the information extraction of medicinal plant growing zones using remote sensing satellite data [2,3,4,5]. However, these studies used limited band information, while the red edge, as a vegetation-sensitive band, is more effective in reflecting the spectral characteristics of *E. ulmoides* [6,7]. Therefore, the extraction of medicinal plants based on the red-edge band of satellite images is an important research direction. The Chinese GF-6 Wide Field of View camera (GF-6 WFV) has a spatial resolution of 16 m and a revisit period of 4 days. Compared to the earlier Chinese GF-1 satellite, the GF-6 satellite adds a purple band, a yellow band, and two red-edge bands, making it sensitive to unique biochemical or structural features of crop types [8]. In particular, previous studies have assessed the importance of red-edge bands of the GF-6 data in crop-type mapping [9]. Therefore, studying the method of identifying the distribution area of *E. ulmoides* using the GF-6 red-edge band and its derived red-edge vegetation index is the basis for its carbon sequestration capacity assessment and ecological suitability area prediction.

Net primary production (NPP) is the amount of biomass or carbon produced by primary producers per unit area and time, obtained by subtracting plant respiratory costs (Rp) from gross primary productivity (GPP) or total photosynthesis. It is the material basis for the survival and reproduction of other food chain members in the ecosystem, an important indicator of the carbon sequestration capacity of plants, and a major factor in determining the carbon sink of terrestrial ecosystems [10,11,12]. Direct in situ measurements are time-consuming and laborious, so simulation models are generally used to analyze NPP [13]. The Carnegie Ames Stanford Approach (CASA) to the light energy utilization model is an effective process-based model for assessing carbon uptake via regional ecosystems based on the acquisition of regional NDVI data, vegetation type maps, and monthly weather data (total solar radiation, temperature, and precipitation) using a combination of remote sensing and GIS techniques to describe carbon exchange between the terrestrial biosphere and atmospheric processes; it has been widely used to model regional or continental NPP in a large number of published studies [14,15].

The potential distribution zone of *E. ulmoides* is predicted, which is conducive to the rational layout, the scientific introduction of *E. ulmoides* resources, and the enhancement of ecological benefits. The niche model can be used to assess and predict the effect of climate change on plants. Currently, several models, such as bioclimatic modeling (BIOCLIM), global geographic information system for a medicinal plant (GMPGIS), climate change experiment (CLIMEX), genetic algorithm for rule-set production (GARP), and maximum entropy (MaxEnt) have been used to predict the potential distribution of species [16,17,18,19,20]. Among these, the MaxEnt-based model is a frequently used tool. The theory of maximum entropy was first proposed in 1957 [21]. The Java MaxEnt model, which was developed from this theory, has become the most commonly used species distribution model (SDM) [21]. It can assess the potential distribution of diseases and insect pests and reasonably predict zones where disease symptoms may occur under climate change conditions [22]. The MaxEnt model has been used in research on natural reserve design, endangered species survey, alien species risk assessment, and climate change impact [23,24,25].

In summary, the main objective of this study was to assess the potential variables of GF-6 data and construct different models to determine the distribution area of *E. ulmoides* based on reliable field survey data. On this basis, the carbon sequestration capacity of *E. ulmoides* was assessed, and its ecological suitability zoning was predicted to provide data support for its sustainable utilization and rational layout. First, optical features, especially the red-edge spectral bands and their normalized vegetation indices (NDVI), were extracted from the GF-6 multi-temporal images and used as input features for the classification of *E. ulmoides*. By comparing the results of the random forest algorithm with different feature combinations to obtain the best feature combination for *E. ulmoides* type discrimination. The random forest classifier is applied to the best set of features to generate a map of *E. ulmoides* distributions. Second, the CASA model was used for the estimation of the NPP of *E. ulmoides* for its carbon sequestration capacity. Third, the MaxEnt model was used to predict the distribution probability maps of the ecological suitability of *E. ulmoides* under current and future climatic conditions. This study aims to answer the following questions: (1) Can the inclusion of red-edge spectral bands and their normalized vegetation indices improve the accuracy of *E. ulmoides* identification? Which spectral bands can be more effective for *E. ulmoides* identification? (2) What is the carbon sequestration capacity of *E. ulmoides*? Does it have high ecological benefits? (3) If the artificial cultivation of *E. ulmoides* is developed, how are its ecological suitability zones distributed? How should planting planning be carried out?

## 2. Materials and Methods

### 2.1. Study Area

**Figure 2:**
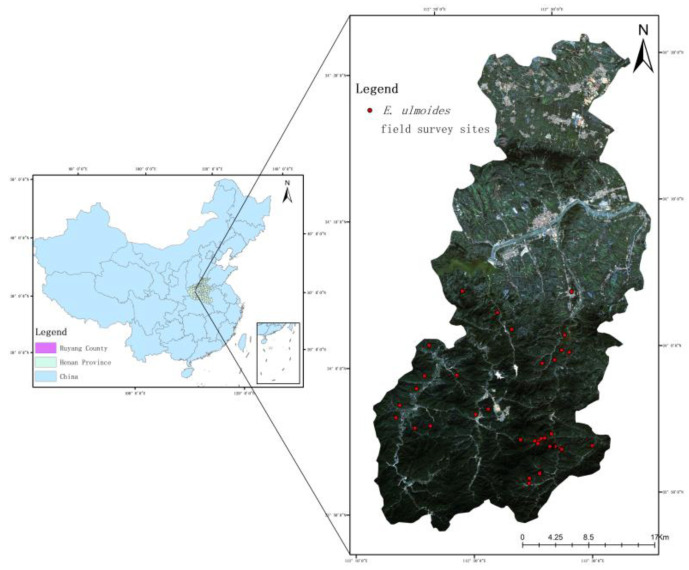
Geographical location and satellite images of Ruyang County.

Ruyang County is located in the western part of Henan Province, at longitude 112°8′~112°38′ E and latitude 33°49′~34°21′ N. The total area is 1325 km^2^, of which more than 900 km^2^ is mountainous, accounting for 70.2% of the total area [11]. The topography of the study area is complex, with continuous mountainous ranges in the south and plains and hills in the north. The ambiguous temperate continental monsoon climate makes the study area well-lit, with a mild climate and four distinct seasons. The average annual sunshine hours are 2177.3 h, the sunshine percentage reaches 49%, the average annual temperature is 14 °C, the average annual rainfall is 690 mm, and the annual frost-free period is 213 days. The location of the study area and GF-6 images are shown in Figure 2.

### 2.2. Data 

#### 2.2.1. In Situ Reference Data

Ground samples of four land cover types (cropland, other forests, urban area, and waterbody) were visually identified from Google Earth high-resolution images in terms of polygons. These sample polygons were resampled to generate randomly placed points for each cover type (Table 1). The individual fields of *E. ulmoides* were collected in a field campaign in July 2020. *E. ulmoides* sample points were randomly extracted from each field under the condition that the minimum distance between any two points must be no less than 20 m. The number of sample points for the five land cover types is shown in Table 1. These samples are divided into training and testing sets in the ratio of 3:1.

#### 2.2.2. Satellite Data Preprocessing and Feature Extraction

This study used satellite image data from the China Centre for Resources Satellite Data and Application (https://data.cresda.cn/ (accessed on 6 July 2023)) Gao Fen-6 (GF-6) and the Wide Field of View (WFV) satellite images, with a width of 800 km. In addition to the common blue, green, red, and near-infrared bands, the red-edge band, which can effectively reflect the unique spectral characteristics of crops, was added for the first time (Table 2), which can provide more detailed spectral information for vegetation studies. Nine images were acquired using the WFV sensor of the GF-6 satellite on 9 May, 28 June, and 9 September 2021. 

Similarly, the 20 monthly GF-6 satellite images covering the study area from January to December 2022 were obtained to estimate NPP. These images were all selected when the cloud cover was less than 10% and covered the whole territory of Ruyang County. 

The dates of the selected remote sensing image were mainly based on the following consideration: the trees in the forest grow slowly in two years. The difference between the acquisition time of the sample plot data and the remote sensing data was less than two years, which had little effect on the results.

The acquired GF-6 WFV images were preprocessed with radiometric calibration, atmospheric correction, and ortho-correction. The specific process was as follows:

First, the band DN values of the data were converted to radiance values based on the calibration coefficients provided by the China Centre for Resources Satellite Data and Application (CRESDA), which are given using the following formula:

(1) $$L_{\lambda }=Gain\times DN+bias$$

where $L_{\lambda }$ is the cell value as radiance; Gain is the gain value for a specific band; DN is the cell value digital number; bias is the bias value for a specific band.

Second, atmospheric correction was performed using the FLAASH (Fast Line-of-sight Atmospheric Analysis of Spectral Hypercubus) module, which is designed to remove atmospheric effects.

Third, with the support of the Global Multi-resolution Terrain Elevation Data 2010 (GMTED2010), the ortho-correction was performed, and the error was required to be within 1 pixel.

Apart from the eight bands of the GF-6 WFV, the normalized difference vegetation index (NDVI) and red-edge indices (Table 3) were generated for each GF-6 image.

#### 2.2.3. Vegetation Type Data, Meteorological Data, and MOD17A3HGF Product Dataset

Vegetation-type data were obtained from the National Cryosphere Desert Data Center. The monthly mean temperature and monthly total precipitation were obtained from the National Oceanic and Atmospheric Administration (NOAA) and the National Centers for Environmental Information (NCEI). Monthly total solar radiation data were obtained from NASA Goddard Earth Sciences (GES) Data and Information Services Center (DISC); the MOD17A3HGF product dataset was obtained from the Land Processes Distributed Active Archive Center (LP DAAC) [26].

#### 2.2.4. Nineteen Bioclimatic Variables Data 

Nineteen bioclimatic variables were selected from the WorldClim data website (http://www.worldclim.org/ (accessed on 6 July 2023)) for the ecological suitability area study (Table 4). The near-current climate simulation data were selected for the period 1970–2000 at a resolution of 30 arc seconds. Future climate data were selected from the ssp245 climate scenario (2041–2060) of the BCC-CSM2-MR dataset product at a resolution of 30 arc seconds.

### 2.3. Methods

**Figure 3:**
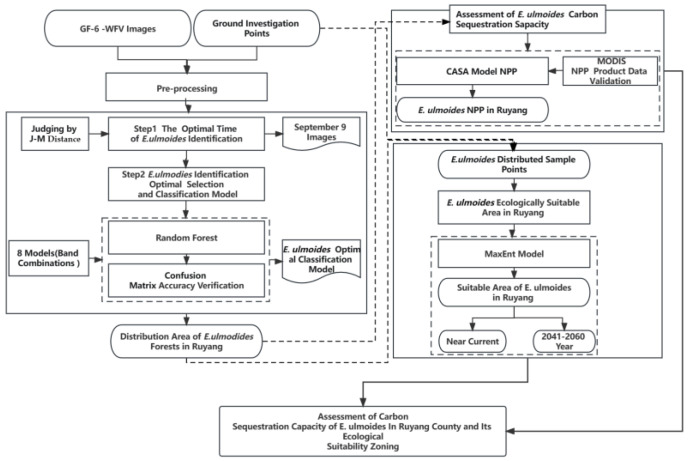
The workflow of assessment of carbon sequestration capacity of *E. ulmoides* in Ruyang County and its ecological suitability zoning based on satellite images of GF-6.

The workflow used in this research is provided in Figure 3. 

In the first step, the optimal time window selection study used the images of the different growing seasons of Ruyang *E. ulmoides*, including the satellite remote sensing images in May, June, and September, and selected the optimal time phase of Ruyang *E. ulmoides* based on different combinations of wave bands and vegetation indexes with the judgment of J–M distance.

The second step was optimal feature selection and classification. The classification method of random forest is used for classification, and by comparing the classification results of different models, the model with the highest precision is selected to extract the regional distribution information of Ruyang County *E. ulmoides*.

The third step was to assess the carbon sequestration capacity of *E. ulmoides*. The CASA model was used to estimate the NPP of Ruyang County, and the results were compared with the MODIS NPP products for verification. Based on the remote sensing extraction results of *E. ulmoides* and the NPP estimation results of Ruyang County, the NPP value of *E. ulmoides* was obtained, which was used as an index to evaluate the carbon sequestration capacity of *E. ulmoides*.

The fourth step was determining an ecologically suitable area for *E. ulmoides* in Ruyang. We combined the results of the remote sensing classification of *E. ulmoides* distribution and *E. ulmoides* survey sample points using the climate variable factors. Based on the MaxEnt model, the study of an ecologically suitable area for *E. ulmoides* in Ruyang County for the contemporary and future years of 2041–2060 was carried out, and the trend of *E. ulmoides* distribution in Ruyang County was predicted to change in the future under the influence of climate. The distribution trend of Ruyang *E. ulmoides* under the influence of future climate was predicted.

Finally, based on the above results, we assessed the carbon sequestration capacity of *E. ulmoides* in Ruyang County and its ecological suitability zoning based on the satellite images of GF-6.

#### 2.3.1. Remote Sensing Identification of *E. ulmoides* Distribution Area

The Jeffries–Matusita (J–M) distance is defined as the ratio of inter-class spectral variability to intra-class spectral variability [27]. Inter-class variability and intra-class variability are used to measure whether the feature set can distinguish different land use types effectively, and the feature set that produces the most internally consistent class and the biggest difference between the different classes is the optimal feature set [28]. In this study, the J–M distance was chosen to determine the spectral separability between feature classes. J–M distance was an important parameter to judge the separability between samples. Using J–M distance analysis, the identification ability of *E. ulmoides* in red-edge band data can be preliminarily judged [29]. The formula to determine the J–M distance is as follows:

(2) $$J_{ij}=2\left(1-e^{-B}\right)$$

(3) $$B=\frac{1}{8}{\left(\mu_{i}-\mu_{j}\right)}^{2}\left(\frac{2}{\sigma_{i}{}^{2}+\sigma_{i}{}^{2}}\right)\left(\mu_{i}-\mu_{j}\right)+\frac{1}{2}\ln\left[\frac{\sigma_{i}{}^{2}+\sigma_{i}{}^{2}}{2\sigma_{i}\sigma_{j}}\right]$$

In the formula, $J_{ij}$ is the J–M distance between category i and category j; B denotes the baroclinic distance of a certain feature dimension; $\mu_{i}$ and $\mu_{j}$ denote the sample means of category i and j on a certain feature, respectively; $\sigma_{i}$ and $\sigma_{j}$ denote the standard deviation of category i and j on a certain feature, respectively. $J_{ij}$ takes a value between 0 and 2, and the larger the value is, the higher the separability of the sample data set in the spectral space and the better the classification result. When the value of $J_{ij}$ is between 1.8 and 2.0, it means that the separability of the sample data set in the spectral space is optimal, and the larger the value, the higher the accuracy of feature classification.

Random forest (RF) [30] is a classifier that trains and predicts samples via an integrated learning approach [31]. This algorithm integrates multiple decision trees and is a highly flexible machine learning algorithm. RF has been widely used for remote sensing classification, as it runs efficiently on large databases and performs well with high-dimensional features. In this study, we used the RF classifier for classification. The RF classifier mainly includes two important parameters: (1) the number of decision trees and (2) the maximum number of features in each tree. Theoretically, the more trees, the better the classification result, but at the same time the classification time increases. Therefore, we need to find a reasonable number of trees. Studies have shown that when the number of trees exceeds 400, the OOB (out-of-bag) error for each classification case tends to stabilize [32]. To ensure the accuracy of classification and minimize the computation time, we set the number of trees to 500. In general, we took the square root of the total number of input features as its value.

The process of using RF for *E. ulmoides* plantation area extraction was as follows: first, sample datasets of different feature types (urban areas, waterbody, cropland, *E. ulmoides*, and other forests) were selected in the remote sensing images of the study area based on the ground interpretation marker point data collected in the field; second, based on the selected sample datasets, the training set was randomly selected, and the decision tree was generated by randomly selected features; finally, RF was used to automatically classify the remote sensing datasets of the study area.

**Figure 4:**
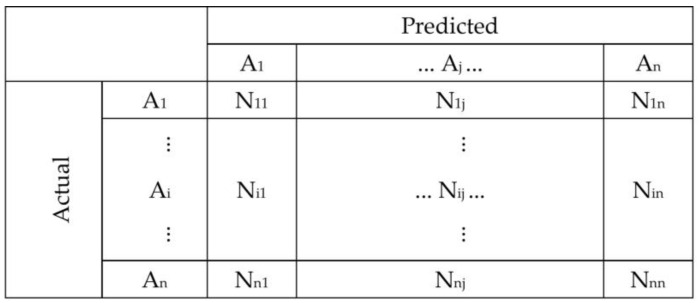
Confusion matrix [33].

A confusion matrix is a concept from machine learning, which contains information about actual and predicted classifications via a classification system. A confusion matrix has two dimensions; one dimension is indexed by the actual class of an object, and the other is indexed by the class that the classifier predicts. Figure 4 presents the basic form of the confusion matrix for a multi-class classification task, with the classes A_1_, A_2_, and A_n_ [33]. 

The accuracy verification of the *E. ulmoides* distribution extraction results is to use the field investigation *E. ulmoides* distribution points as the verification dataset and use the confusion matrix method [34] to select the overall accuracy (OA), Kappa coefficient, user accuracy (UA also referred to as precision), and producer’s accuracy (PA also referred to as recall) as evaluation indexes to evaluate the accuracy of the *E. ulmoides* classification results in Ruyang County. The overall accuracy was calculated as follows:

(4) $$\mathrm{OA}=\frac{\sum_{i=1}^{n}S_{i}\sum_{j=1}^{k}\alpha_{ij}\beta_{ij}}{\sum_{i}^{n}S_{i}}$$

where  n is the total number of segments, k is the number of classes, $\alpha_{ij}$ is 1 if the reference label for segment i is j and 0, and $\beta_{ij}$ is 1 if the map label for segment i is j. The true classification of segment i is indicated as $\sum_{j=1}^{k}\alpha_{ij}\beta_{ij}$ = 1, where the reference and map classes for segment i are equal. For area-based metrics, $S_{i}$ is the area of segment i. For count-based metrics, $S_{i}$ is 1.

The producers’ accuracy (recall) indicates the probability that an object belonging to class j is correctly classified as follows:

(5) $${\mathrm{PA}}_{j}=\frac{\sum_{i=1}^{n}S_{i}\alpha_{ij}\beta_{ij}}{\sum_{i=1}^{n}\alpha_{ij}S_{i}}$$

The users’ accuracy (precision) indicates the probability that an object mapped as belonging to class j does actually belong to that class as follows:

(6) $${\mathrm{UA}}_{j}=\frac{\sum_{i=1}^{n}S_{i}\alpha_{ij}\beta_{ij}}{\sum_{i=1}^{n}\beta_{ij}S_{i}}$$

The F1 score is a more valuable measure than OA in the case of an unequal distribution of categories, as it is a weighted average of precision and recall. The F1 score for each class is calculated as follows:

(7) $$F1=2\frac{\mathrm{precision}\times \mathrm{recall}}{\mathrm{precision}+\mathrm{recall}}$$

#### 2.3.2. Assessment of *E. ulmoides* Carbon Sequestration Capacity

CASA is a light energy utilization model driven by a combination of remote sensing, meteorological, vegetation, and soil type data [35,36]. NPP is mainly determined using two variables absorbed via vegetation: Absorbed Photosynthesis Active Radiation (APAR) and light energy utilization (ε). Therefore, NPP can be expressed as the following equation [37,38]:

(8) $$\mathrm{NPP}\left(x,t\right)=\mathrm{APAR}\left(x,t\right)\times \varepsilon \left(x,t\right)$$

In the formula, APAR and ε are calculated using the following formula:

(9) $$\mathrm{APAR}\left(x,t\right)=0.5\times \;\mathrm{SOL}\left(x,t\right)\times \;\mathrm{FRAR}\left(x,t\right)$$

(10) $$\varepsilon \left(x,t\right)={\;T}_{\varepsilon 1}\left(x,t\right)\times T_{\varepsilon 2}\left(x,t\right)\times W_{\varepsilon }\left(x,t\right)\times \varepsilon_{max}$$

where SOL $\left(x,t\right)$ represents the total solar radiation at the pixel x of t month (MJ·m^−2^ month^−1^).

In the formula, SOL $\left(x,t\right)$ represents the total solar radiation (MJ·m^−2^ month^−1^) at the pixel x of t month; FRAR $\left(x,t\right)$ represents the absorption ratio of the photosynthetic active radiation of the sun incident by the vegetation layer; 0.5 refers to the proportion of the solar effective radiation used by the vegetation in the total radiation; $\varepsilon \left(x,t\right)$ is the efficiency with which plants convert absorbed photosynthetically active radiation into organic carbon; $T_{\varepsilon 1}\left(x,t\right)$ $\left(x,t\right)$ is the extent to which the net primary productivity of plants is reduced due to the restriction of photosynthesis caused by the physiological effects of plants at low and high temperatures; $T_{\varepsilon 2}\left(x,t\right)$ $\left(x,t\right)$ showed a decreasing trend of energy utilization when the optimum growth temperature changed to high and low temperature; $W_{\varepsilon }\left(x,t\right)$ $\left(x,t\right)$ is the limit degree of water state on light energy use efficiency; $\varepsilon_{max}$ is the maximum light energy utilization under ideal conditions [39].

In this study, we used the NPP estimation program developed by Zhu Wenquan et al. [39] based on the CASA model, which was installed in the ENVI (The Environment for Visualizing Images) software. The NPP estimation program has input boxes such as static parameter files, vegetation type maps, NDVI time series, monthly mean temperature, monthly total precipitation, and monthly total solar radiation, as well as output paths. The static parameter files are the maximum and minimum values of NDVI, the maximum and minimum values of SRVI, and the maximum light energy utilization corresponding to each vegetation type, which can be obtained by searching for the relevant regional literature or automatically configured via the NPP software based on the NDVI time series corresponding to each vegetation type, and the method chosen in this study is the automatic configuration. The pixel size and projection of the vegetation type data should be the same as that of the NDVI data. The NDVI was obtained by preprocessing the downloaded 12-month remote sensing images with ENVI and then using the NDVI calculation function. Meteorological data were processed by downloading the 12 month monthly mean temperature, monthly total precipitation, and monthly total solar radiation data from the weather stations, interpolating the meteorological data into kriging space using ArcGIS to form spatially continuous month-by-month climate change data, and obtaining the raster image of the meteorological data that was also identical to the pixel sizes and projections of the NDVI data. Finally, these data were input into the CASA model to estimate the NPP of the study area.

The validation of NPP can be carried out using two methods: the measured value validation method and the relative validation method. Due to the lack of measured data, this study used the indirect validation method to compare the CASA model NPP-inversion results with MOD17A3HGF NPP data. The MOD17A3NPP product dataset was obtained by Running et al. [40] using the BIOME-BGC model (biome biogeochemical model) and light energy utilization model simulation. Currently, this product can estimate the spatial and temporal changes in NPP in global ecosystems and has been validated and widely used in the evaluation of different vegetation growth conditions, environmental monitoring, and carbon cycle studies. Therefore, in this study, 30 points were randomly generated in the study area, and the MOD17A3HGF NPP values and the NPP results estimated using the CASA model were extracted from these 30 points to validate the model’s estimation of Ruyang County’s NPP. The root mean square error (RMSE) and coefficient of determination (R^2^) were used in this study to measure the accuracy of the CASA model [41].

(11) $$RMSE=\sqrt{\frac{\sum {\left(Y_{t}-Y_{p}\right)}^{2}\;}{N}}$$

(12) $$R^{2}=\frac{Cov\left(Y_{t},Y_{p}\right)}{\sigma Y_{t}\sigma Y_{p}}$$

where $Y_{t}$ is the MOD17A3 NPP value for the Nth *E. ulmoides* study site, and $Y_{p}$ is the NPP value estimated using the CASA model for the Nth *E. ulmoides* study site.

#### 2.3.3. *E. ulmoides* Ecologically Suitable Area in Ruyang

Species distribution models (SDMs) are used extensively in the field of landscape ecology and conservation biology since their origin in the late 1980s. But, there is still a void in the field, i.e., a universal modeling approach for SDMs. MaxEnt is a widely expected algorithm due to its robust and nonlinear modeling techniques [42]. MaxEnt models can satisfy all known variables without any subjective assumptions, which is not present in earlier SDM models (such as Bioclim/DOMAIN) [43]. A study attempted to establish the relation between species occurrence data and their respective environmental predictor variables. The yearly trend of each parameter was analyzed to observe the variations throughout the year, and a pixel-wise mean value was calculated to be used in the SDM. Machine learning algorithms, namely MaxEnt, boosted regression trees (BRT), RF, and generalized linear model (GLM), were implemented to establish the relationship between predictor variables. The AUC-based performance evaluation metric was generated, and it was found that MaxEnt performs better than others. The model is more tolerant of small samples, irregular samples, and site bias data and is preferred for predicting the potential distribution zone of rare species [44,45].

The geographic distribution points and environmental variables of *E. ulmoides* were imported into the MaxEnt 3.4.1 software for modeling operations. In total, 75% of the distribution data were randomly selected as the training set and 25% as the test set. The maximum number of iterations was set to 1000, and Bootstrap was selected to create different sets of test and validation numbers for each iteration. The calculation was repeated 10 times, and the jack-knife method was turned on to calculate the effects of the environmental variables on the distribution of *E. ulmoides*, and the response curves of each environmental variable were plotted. The results were output in logistic form, the raster values were survival probabilities (*p*-values), and the output file was in .asc format.

The prediction results of the model were evaluated using the range of area under curve (AUC) values. The AUC value was the area under the receiver operating characteristic (ROC) curve plotted with specificity as the horizontal coordinate and sensitivity as the vertical coordinate. The value of AUC ranged from 0 to 1. A larger value indicated that the farther away it is from the random distribution, the better the prediction.

## 3. Results

### 3.1. Results of E. ulmoides Distribution in Ruyang

#### 3.1.1. The Optimal Time for *E. ulmoides* Identification

In this study, J–M distances were calculated based on six bands excluding the red edge (combination 1: B1–B4, B7, and B8), all eight bands including the red edge (combination 2:B1–B8), and all eight bands plus three bands of NDVI, NDVI_710_, and NDVI_750_ vegetation indices (combination 3:B1-B8, NDVI, NDVI_710_, and NDVI_750_).

To analyze the influence of red-edge bands on the separability and recognition ability of *E. ulmoides*, the J–M distances (separability) of the typical features in the study area were calculated for three combinations of bands at different time phases using the same sample data set, as shown in Table 5. As seen in Table 5, after increasing only the red-edge band, the J–M distance between the three time-phase images of *E. ulmoides* and other forests was still less than 1.8; whereas after increasing both the red-edge band and the three vegetation indices, the J–M distance between the three time-phase images of *E. ulmoides* and other forests increased to more than 1.8, among which the J–M distance between *E. ulmoides* and other forest in the 9 September image reached 1.866 with the best separable ability.

In summary, the introduction of a red-edge band and vegetation index can increase the separability of *E. ulmoides*, and the best separability of *E. ulmoides* from other features was observed in the September 9 image.

#### 3.1.2. *E. ulmoides* Identification Optimal Selection and Classification Model

Table 6 shows that model 1, which is the model without the participation of a red-edge band and vegetation index in the classification, has the lowest OA, Kappa coefficient, and UA. Model 7 is the model with the participation of red-edge band 6 and its derived red-edge vegetation index NDVI_750_ in the classification, and the OA, Kappa coefficient and PA are the highest, indicating that the red-edge band information can help the images distinguish *E. ulmoides* from other features more accurately.

Models 2, 3, and 4 were added with different red-edge bands to participate in the extraction of the *E. ulmoides* distribution zone, and the results showed that the OA of model 4 increased by 0.56% compared with models 2 and 3. Meanwhile, the PA of model 4 increased by 1.14% and 4.93% compared with models 2 and 3. These results indicate that model 4 outperforms models 2 and 3 and further shows that the model with the joint participation of band 5 and band 6 in the red-edge band is better than the model with only band 5 or band 6 participation in the model. The overall recognition of features is better, and the omission error of dulcimer is smaller. However, model 3 has the highest UA compared to models 2 and 4, indicating that the model with only red-edge band 6 participation has fewer misclassification errors for *E. ulmoides*. 

Models 5, 6, 7, and 8 were added with different vegetation indices involved in the extraction of the *E. ulmoides* distribution zone, and the results showed that model 7 outperformed models 5, 6, and 8 in terms of OA, Kappa coefficient, and PA, indicating that the model involving red-edge band 6 and its derived red-edge vegetation index NDVI_750_ had the strongest ability to identify *E. ulmoides*.

**Figure 5:**
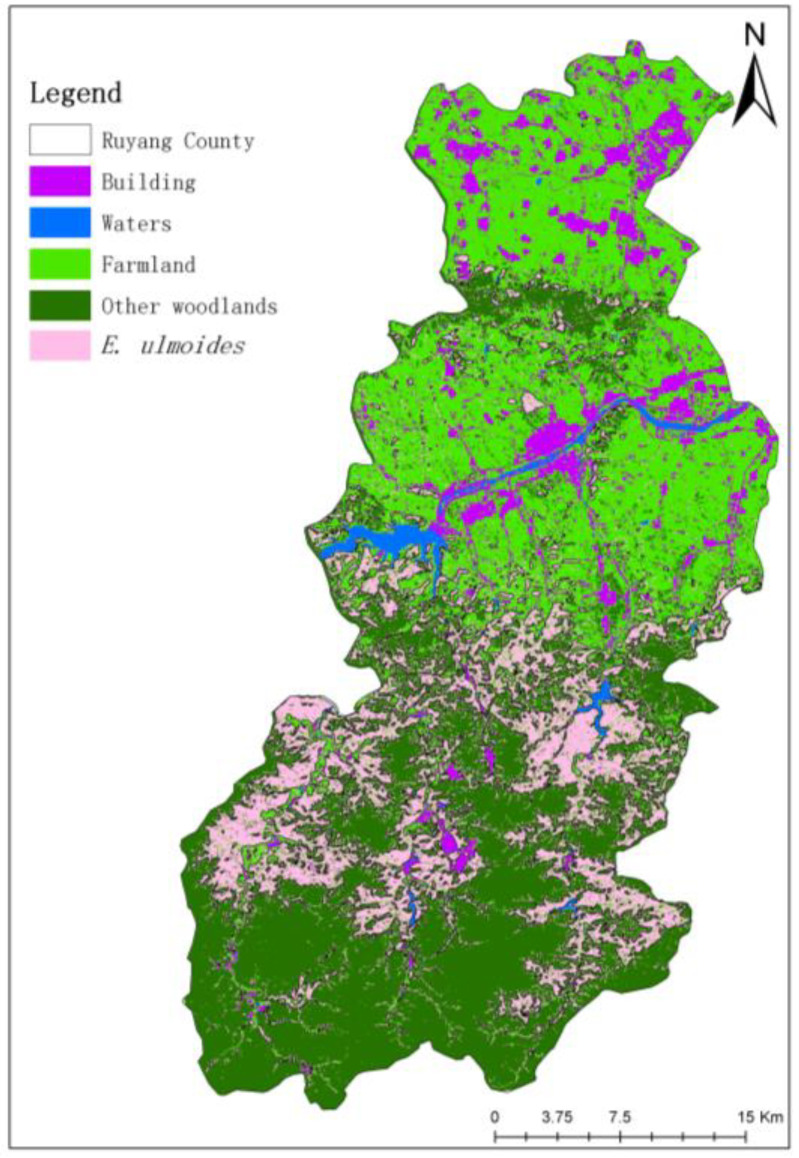
Classification results of 5 typical features in Ruyang County.

According to the above analysis, based on the single temporal phase GF-6 WFV remote sensing image of Ruyang County on 9 September, model 7 was used to extract the classification of *E. ulmoides* in the County, and the results are shown in Figure 5. The classification results were evaluated for accuracy using the confusion matrix, and the results are shown in Table 7.

As seen in Figure 5, the *E. ulmoides* planting area is mainly distributed in the mountainous zone in the south-central part of Ruyang County, including the three towns of Shangdian, Santun, Fudian, and the three townships of Shibapan, Jincun, and Wangping. 

As seen in Table 7, the results of the evaluation of the classification accuracy of *E. ulmoides* were 96.62% for the OA, 0.89 for the F1 score, and 92.00% for the PA, which indicates 8.00% for the misclassification error, and 86.56% for the UA, which indicates 13.44% for the omission error. 

### 3.2. Assessment Results of Carbon Sequestration Capacity of E. ulmoides in Ruyang County

**Figure 6:**
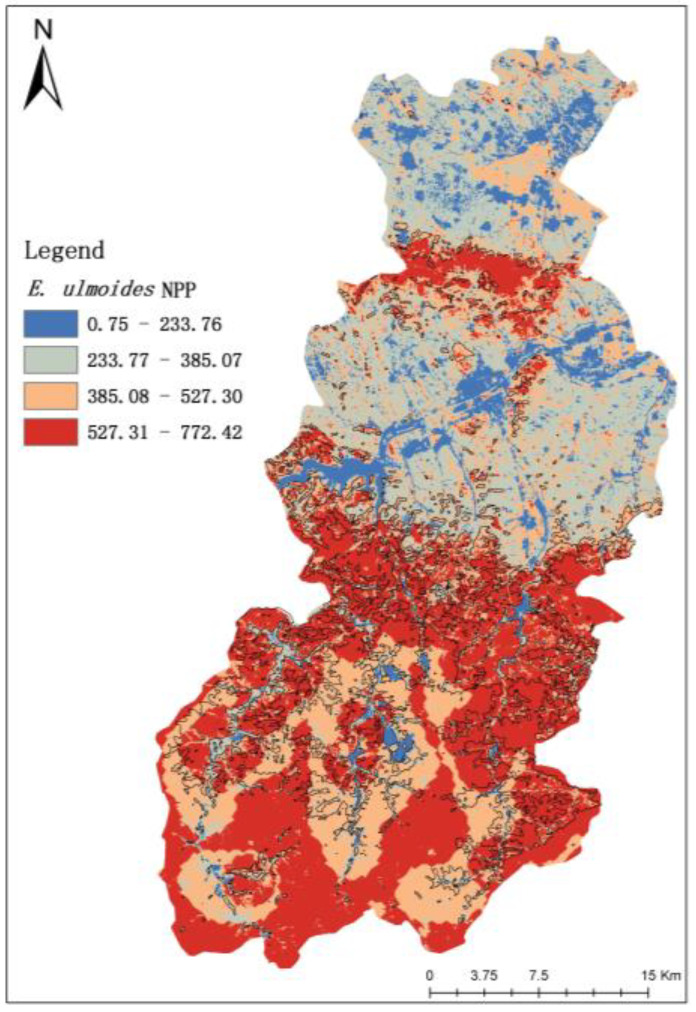
Results of NPP estimation of Ruyang County (include *E. ulmoides*).

The NPP results of Ruyang County in 2022 were estimated based on the CASA model, which was divided into four grades using the natural break point method in the Geographic Information System (GIS). They were low (0.75~233.76 g CM^−2^·a^−1^), medium (233.77–385.07 g CM^−2^·a^−1^), high (385.08–527.30 g CM^−2^·a^−1^), and very high (527.31–772.42 g CM^−2^·a^−1^). The results are shown in Figure 6. The NPP low-value area accounts for 9.25% of the area of Ruyang County and is mainly distributed in the southern and south-central regions. According to the classification results of remote sensing images, this area is mainly waterbody and urban areas. The NPP median value area is distributed in the south and south-central part of Ruyang County, but its land type is cropland, and its area is larger than the low-value area, accounting for 25.13% of the area of Ruyang County. 

The NPP higher value area is scattered in the south of Ruyang County. It is concentrated in the north of Ruyang County, and its land use type is also different, the south being dominated by cultivated land, while the north is mainly other forests. The total area accounts for 30.67% of the area of Ruyang County. The NPP high-value area in the north of Ruyang County shows a large concentration, the south with a small concentration, and the land use type is dominated by other forests and *E. ulmoides* plantation area, accounting for 34.95% of the area of Ruyang County.

**Figure 7:**
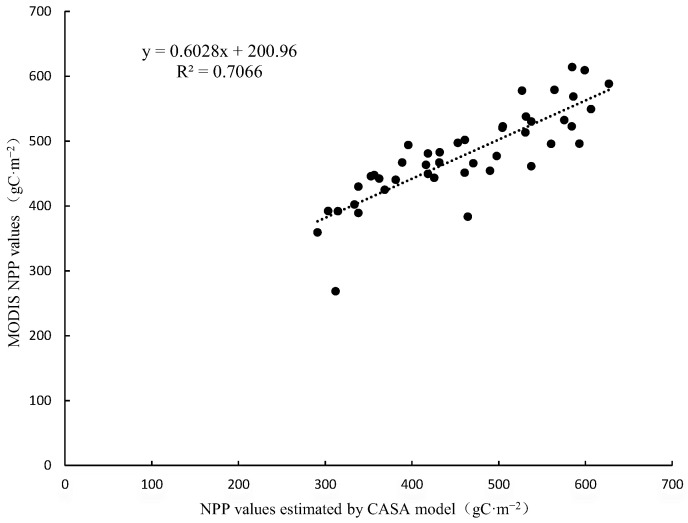
Relationship between MODIS NPP values and NPP values estimated using CASA model.

Based on the comparison results of NPP values and land use types, this result is more reasonable. The accuracy of the data was further evaluated in this study, which compared the NPP values obtained using the MODIS NPP product and the CASA model and showed an RMSE of 56.4550 and a correlation coefficient R^2^ of 0.70669 (Figure 7). It indicates that the results of NPP estimation using the CASA model have a good correlation with the results of the MOD17A3HGF product dataset. It further indicates that the accuracy of the NPP results estimated using the CASA model in this study is high, and it can be considered that the CASA model of light energy utilization applies to the study of NPP estimation in the *E. ulmoides* plantation area in Ruyang County. In addition, the high-value area of NPP of *E. ulmoides* in Ruyang County was mainly distributed in the south-central part of Ruyang County, and the annual total NPP carbon sequestration in the distribution area of *E. ulmoides* was estimated to be 540.104835 t CM^−2^·a^−1^ based on the CASA model.

### 3.3. Results of Ecological Suitability Area

**Figure 8:**
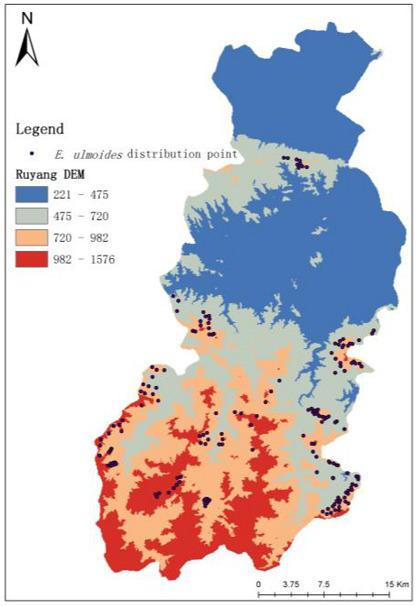
Distribution of *E. ulmoides* samples in Ruyang County.

**Figure 9:**
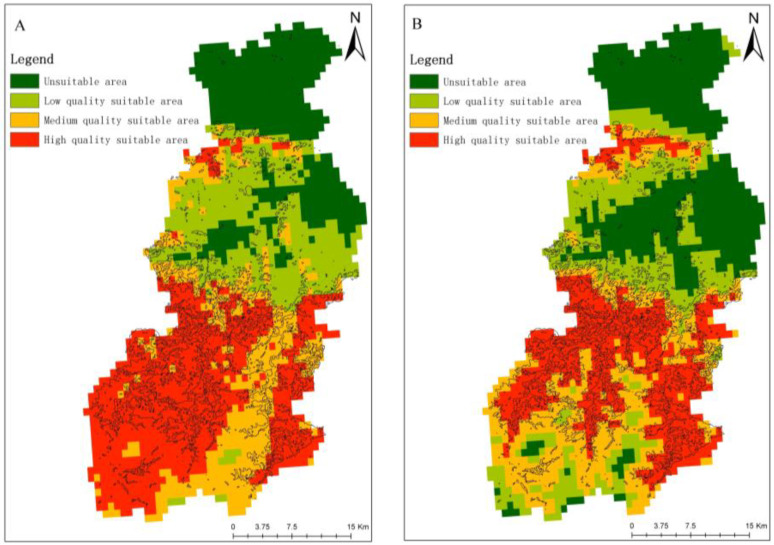
Distribution of suitable area of *E. ulmoides* in Ruyang County under current and future climate conditions ((**A**) current; (**B**) 2041–2060).

**Figure 10:**
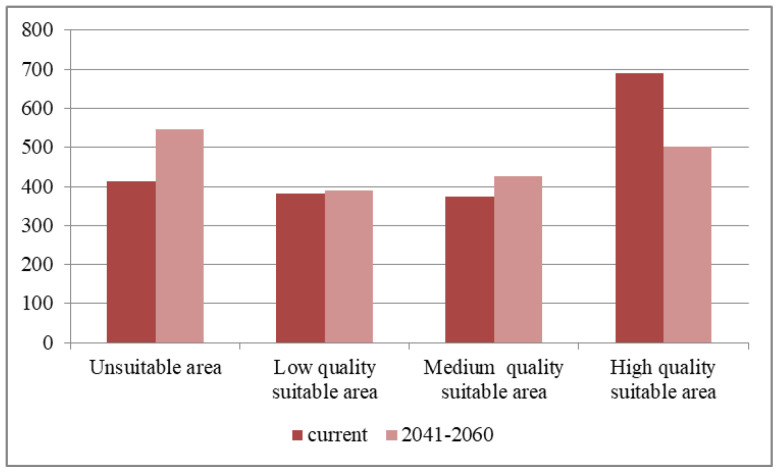
Area of ecologically suitable zone of *E. ulmoides* in Ruyang County under current and future climatic conditions.

The sample points of the input MaxEnt model *E. ulmoides* are shown in Figure 8. The output results of the MaxEnt model were reclassified in ArcGIS10.8, and the classification method was the natural break method, which was divided into four classes: 0–0.16 for the non-suitable area, 0.16–0.35 for the low suitable area, 0.36–0.5 for the medium suitable area, and 0.51–0.7 for the high suitable area. The number of pixels of different fitness zones was counted with a spatial resolution of 1 km, and the area of different fitness zones was calculated by multiplying the number of pixels by the spatial resolution. The results are shown in Figure 9 and Figure 10.

As seen in Table 8, the climatic factors affecting the distribution of *E. ulmoides* in Ruyang County with higher contribution are the driest seasonal precipitation at 29.3%, the seasonal variation in temperature at 16.2%, the isothermal temperature at 15.7%, and the average monthly temperature variation range at 12.5%. The replacement importance is greater for the driest season precipitation 82.6%, and the coldest season average temperature is 6.9%.

**Figure 11:**
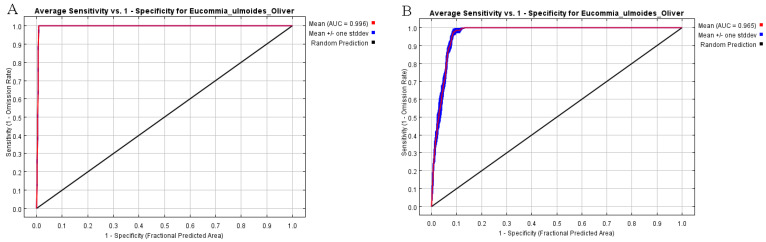
ROC curves of simulated distribution results of *E. ulmoides* under current and future climatic conditions ((**A**) current; (**B**) 2041–2060).

As shown in Figure 11, the ROC curve shows that AUC = 0.996 for Ruyang County *E. ulmoides* in the current climate environment, indicating that the MaxEnt model prediction results were extremely accurate and credible. In the future (2041–2060), the ROC curve shows that AUC will be 0.965, indicating that the MaxEnt model prediction results were extremely accurate and reliable.

The climatic factors affecting the distribution of *E. ulmoides* in Ruyang County mainly include precipitation in the driest season, temperature seasonal standard deviation, iso-temperature, daily mean temperature range, and average temperature in the coldest season. 

Under the contemporary climate environment, the low suitable area of *E. ulmoides* in Ruyang County is 383 km^2^, the middle suitable area is 375 km^2^, the high suitable area is 691 km^2^, and the unsuitable area is 414 km^2^. In future climates (2041–2060), the low suitable area of *E. ulmoides* Ruyang County will be 389 km^2^, the middle suitable area will be 427 km^2^, the high suitable area will be 501 km^2^, and the unsuitable area will be 546 km^2^. Compared with the contemporary climate conditions and the future climate conditions, the area of the suitable area of Ruyang County *E. ulmoides* showed a reduction.

## 4. Discussion

The main objective of this study was to assess the potential variables of GF-6 data and construct different models to determine the distribution area of *E. ulmoides* based on reliable field survey data. On this basis, the carbon sequestration capacity of *E. ulmoides* was assessed and its ecological suitability zoning was predicted to provide data support for its sustainable utilization and rational layout. It is particularly important to use remote sensing imagery to establish an identification model of *E. ulmoides* to estimate and map the distribution area of *E. ulmoides*. One of the reasons for this is that *E. ulmoides* is not only a valuable traditional Chinese medicine but also a source of rubber; not only can it be used as a medicinal resource, but it can also be used as an industrial raw material. It is also an important part of China’s forestry industry. Based on this study, we can effectively utilize the GF-6 remote sensing data and RF classification algorithm to spatially map the distribution area of *E. ulmoides*. In addition, the assessment of the carbon sequestration capacity of *E. ulmoides* reveals that *E. ulmoides* has the advantages of a large carbon sink, low cost, and high ecological added value, which makes us realize the great potential of *E. ulmoides* in ecological conservation. Therefore, the key to increasing the storage capacity and carbon sink of *E. ulmoides* lies in vigorously developing the artificial cultivation of *E. ulmoides*, and the study of *E. ulmoides* ecologically suitable zoning can clarify the suitable zones of *E. ulmoides* in the present and future climatic conditions and provide data support for the selection of artificial cultivation areas of *E. ulmoides*.

Based on the spectral bands and vegetation indices extracted from GF-6 data, this study has shown that the red-edge band was the more important variable when estimating the distribution area of *E. ulmoides* using the RF method, which had been confirmed in recent studies concerning red-edge band [46] and crop species classification [47]. In the gross primary productivity field (GPP), Lin et al. found that the red-edge band was useful for estimating the GPP and noted that the red-edge reflectance was sensitive to the leaf chlorophyll content [48]. Therefore, the red-edge band of GF-6 is an important variable in the study of *E. ulmoides* identification. Although the methodology used to study the identification of *E. ulmoides* planting areas obtained the optimal feature sets of *E. ulmoides* and achieved good classification results, some limitations of this study should be noted. First, this study used J–M distances when calculating pairwise separability. Other indicators could be used to quantify the inter-class separability as well. Second, due to the ruggedness of the mountainous terrain and the inaccessibility of some areas, the distribution of the *E. ulmoides* sample points from the field survey was uneven, and the sample data were lacking, which made this approach still unable to avoid the modeling bias caused by the uneven distribution of the data values of the sample. Third, the feature set used for classification in this study involves only the spectral features of the image, not the texture features or other index features of the image. Subsequently, a classification model will be constructed based on a variety of feature sets of the image, and comparisons will be made to study the effect of other features, such as texture, on the results of the recognition of *E. ulmoides*. Finally, although the RF algorithm has been shown to provide excellent performance, the phenomena of the overestimation of low values and the underestimation of high values always exist. The innovation of the algorithms to address this problem will be a future research direction.

As ecological problems become more and more prominent, people pay more and more attention to the carbon sequestration capacity of vegetation. NPP is an important indicator to reflect the carbon sequestration capacity of vegetation. Due to the limitations, it is usually difficult to realize in situ measurements of NPP at large scales. Among the many models for NPP estimation, the CASA model has been widely used in large-scale studies worldwide because it integrates the effects of different natural factors on vegetation NPP [49]. In this study, the 16 m GF-6 WFV image was utilized for the estimation of NPP in Ruyang County instead of the MODIS image (1 km) that is commonly used in the literature, and the results were compared with the MOD17A3HGF product data. The distribution of NPP in Ruyang County shows that it is basically consistent with the distribution of land cover types in Ruyang County. The NPP values of the land cover types were ranked as other forests > *E. ulmoides* > cropland > waterbody ≈ urban areas. In addition, a high correlation can be seen based on the comparison of MOD17A3HGF data with CASA estimates under randomized sample points. Therefore, the results of this study are worthy of being trusted, which, in turn, proves that the CASA model can be used with high-resolution data to calculate the NPP for the county and smaller scales of the study area. However, it is very unfortunate that the actual NPP was not measured, so it was not possible to compare it with the model estimates of NPP. In the follow-up study, we will collect the measured data to compare whether the estimation of NPP with different resolutions using the CASA model in small-scale space will affect the estimation results and how reasonable is the spatial resolution and other extensible issues to provide a reference for the optimization of the CASA model.

In this study, the MaxEnt model predicts the results of the suitable distribution area of *E. ulmoides*, which is mainly affected by the points of the *E. ulmoides* subsample, and the bias of the sample may result in the bias of the results.

Using the *E. ulmoides* classification results to clarify the *E. ulmoides* distribution information has the following advantages for the ecological study of *E. ulmoides*: 1. It is not limited to the *E. ulmoides* distribution point information in the current database. It can be targeted to research a certain region. 2. Good data include timeliness, satellite remote sensing image data availability, and timely data that can also provide timely information on the distribution of *E. ulmoides.* This study can provide a new way of conducting ecological research on a larger scale of *E. ulmoides* in the future.

## 5. Conclusions

In all three time phases, the J–M distances between *E. ulmoides* and the remaining four features increased correspondingly with the involvement of the red-edge band compared to the no-red-edge band, and the separability was enhanced. The J–M distance between *E. ulmoides* and the other four surface features was the largest, and the separability was the strongest when the red band and vegetation index were involved together. When the red edge band and vegetation index were involved together, it was found from the relative ratio of three times that on 9 September, *E. ulmoides* and other features had the greatest separability. Therefore, 9 September is the best time for *E. ulmoides* extraction. In terms of classification accuracy, the OA, Kappa coefficient, and *E. ulmoides* UA were the lowest when no red-edge band and vegetation index were involved in the classification. When the red-edge band 6 and its derived red-edge vegetation index NDVI_750_ participated in the classification, the OA, Kappa coefficient and *E. ulmoides* PA were the highest, which were 96.62%, 0.953, and 92.00%, respectively, indicating that the red-edge band information could make the image more accurately distinguish *E. ulmoides* and other features.

The comparison of *E. ulmoides* NPP results with MODIS NPP product values shows that the CASA model is not only suitable for NPP estimation in Ruyang County but also has high accuracy. Based on the distribution of suitable planting zones for *E. ulmoides* in the north and south of Ruyang County and the current and future distribution, it is suggested that relevant departments should gradually focus on the planting area of *E. ulmoides* in the central and southern parts of Ruyang County, where climatic factors such as precipitation in the driest season, seasonal changes in temperature, iso-temperature and average monthly temperature are more suitable, which is conducive to increasing *E. ulmoides* yield. Moreover, it is more conducive to a more effective combination of agricultural production zones and plant carbon sequestration in the future climate and provides a reference for relevant agricultural management departments to optimize the industrial layout and guide the development of advantageous industries in townships.

## Figures and Tables

**Table 1:** Ground samples of the five land cover types in the study area.

Land Cover Type	Sample Points
Cropland	43
Forest	53
Urban area	45
Waterbody	28
*E. ulmoides*	39

**Table 2:** Spectral band information of GF-6 WFV images.

Band Number	Wave Band	Wavelength Range/nm	Spatial Resolution
B1	Blue	450~520	16 m
B2	Green	520~590	
B3	Red	630~690	
B4	Near-infrared	770~890	
B5	Red Edge 1	690~730	
B6	Red Edge 2	730~770	
B7	Violet	400~450	
B8	Yellow	590~630	

**Table 3:** Spectral indices calculated from GF-6 data.

Abbreviations	Reference Spectral Indices	Formula
NDVI	Normalized Difference Vegetation Index	$\mathrm{NDVI}=\frac{B4-B3}{B4+B3}$
NDVI_710_	Normalized Difference Vegetation Index red-edge at 710 nm (center wavelength)	${\mathrm{NDVI}}_{710}=\frac{B5-B3}{B5+B3}$
NDVI_750_	Normalized Difference Vegetation Index red-edge at 750 nm (center wavelength)	${\mathrm{NDVI}}_{750}=\frac{B6-B3}{B6+B3}$

**Table 4:** Detailed explanation of bioclimatic variables.

Bioclimatic Variables	Description
BIO1	Annual Mean Temperature
BIO2	Mean Diurnal Range (Mean of monthly (max temp–min temp))
BIO3	Isothermality (BIO2/BIO7) (×100)
BIO4	Temperature Seasonality (standard deviation × 100)
BIO5	Max Temperature of Warmest Month
BIO6	Min Temperature of Coldest Month
BIO7	Temperature Annual Range (BIO5-BIO6)
BIO8	Mean Temperature of Wettest Quarter
BIO9	Mean Temperature of Driest Quarter
BIO10	Mean Temperature of Warmest Quarter
BIO11	Mean Temperature of Coldest Quarter
BIO12	Annual Precipitation
BIO13	Precipitation of Wettest Month
BIO14	Precipitation of Driest Month
BIO15	Precipitation Seasonality (Coefficient of Variation)
BIO16	Precipitation of Wettest Quarter
BIO17	Precipitation of Driest Quarter
BIO18	Precipitation of Warmest Quarter
BIO19	Precipitation of Coldest Quarter

**Table 5:** J–M distances of typical features with and without red-edge bands and vegetation indices at different times.

Time	Type	J–M Distance		
		Combination 1	Combination 2	Combination 3
9 May	Other forests—*E. ulmoides*	1.364	1.522	1.857
	Cropland—*E. ulmoides*	1.997	1.999	1.999
	Urban area—*E. ulmoides*	1.999	1.999	2.000
	Waterbody—*E. ulmoides*	2.000	2.000	2.000
28 June	Other forests—*E. ulmoides*	1.092	1.259	1.816
	Cropland—*E. ulmoides*	1.995	1.999	1.999
	Urban area—*E. ulmoides*	1.999	1.999	2.000
	Waterbody—*E. ulmoides*	1.999	1.999	1.999
9 September	Other forests—*E. ulmoides*	1.459	1.713	1.866
	Cropland—*E. ulmoides*	1.921	1.979	1.997
	Urban area—*E. ulmoides*	1.990	1.995	1.999
	Waterbody—*E. ulmoides*	1.999	2.000	2.000

**Table 6:** Classification accuracy of *E. ulmoides* at different combinations of bands.

Models	Band Combinations	OA (%)	Kappa Coefficient	PA (%)	UA (%)
1	B1+B2+B3+B4+B7+B8	95.72	0.9405	88.00	77.78
2	B1+B2+B3+B4+B5+B7+B8	96.28	0.9483	88.57	82.89
3	B1+B2+B3+B4+B6+B7+B8	96.28	0.9482	84.78	89.14
4	B1+B2+B3+B4+B5+B6+B7+B8	96.43	0.9503	89.71	86.26
5	B1+B2+B3+B4+B7+B8+NDVI	96.15	0.9464	91.43	82.90
6	B1+B2+B3+B4+B5+B7+B8+NDVI+NDVI_710_	96.27	0.9481	90.86	82.38
7	B1+B2+B3+B4+B6+B7+B8+NDVI+NDVI_750_	96.62	0.9529	92.00	86.56
8	B1+B2+B3+B4+B5+B6+B7+B8+NDVI+NDVI_710_+NDVI_750_	96.59	0.9526	89.71	83.96

**Table 7:** Precision evaluation of typical ground features classification in Ruyang County.

Feature Type	Urban Area	Waterbody	Cropland	Other Forests	*E. ulmoides*	Total	UA (%)	F1 Score
Urban area	1053	6	15	0	0	1074	98.04	0.98
Waterbody	16	378	0	0	0	394	95.94	0.97
Cropland	8	0	418	9	10	436	95.87	0.94
Other forests	0	0	22	1276	130	1311	97.33	0.97
*E. ulmoides*	0	0	0	250	1610	1860	86.56	0.89
Total	1077	384	455	1310	1750	3401		
PA (%)	97.77	98.44	91.87	97.40	92.00			
OA (%)	96.62							

**Table 8:** Contribution and replacement importance of variables affecting the distribution of *E. ulmoides* in Ruyang County.

Variable	Percent Contribution (%)	Permutation Importance (%)
BIO17	29.3	82.6
BIO4	16.2	0
BIO3	15.7	1
BIO2	12.5	0.5
BIO10	6.7	0.2
BIO8	4.8	3.3
BIO6	4.7	2.6
BIO15	4.1	1.5
BIO13	2.8	0
BIO12	1.3	0
BIO9	0.6	0
BIO11	0.5	6.9
BIO19	0.3	0.1
BIO14	0.2	0.1
BIO7	0.1	0.9
BIO1	0	0.5
BIO5	0	0
BIO18	0	0
BIO16	0	0

## Data Availability

All the data in the article can be found via the links and no unavailable data.
